# New Immunological Markers in Chromoblastomycosis—The Importance of PD-1 and PD-L1 Molecules in Human Infection

**DOI:** 10.3390/jof9121172

**Published:** 2023-12-07

**Authors:** Italo N. Cavallone, Walter Belda, Caroline Heleno C. de Carvalho, Marcia D. Laurenti, Luiz Felipe D. Passero

**Affiliations:** 1 Institute of Biosciences, São Paulo State University (UNESP), Praça Infante Dom Henrique, s/n, São Vicente 11330-900, Brazil; id.cavallone@gmail.com; 2 Laboratory of Pathology of Infectious Diseases (LIM50), Department of Pathology, Medical School, São Paulo University, São Paulo 01246-903, Brazil; 3 Dermatology Department, Medical School, São Paulo University, Clinics Hospital, São Paulo 05403-000, Brazil; walterbelda@uol.com.br (W.B.J.); caroline_heleno@hotmail.com (C.H.C.d.C.); mdlauren@usp.br (M.D.L.); 4 Institute for Advanced Studies of Ocean (IEAMAR), São Paulo State University (UNESP), Rua João Francisco Bensdorp, 1178, São Vicente 11350-011, Brazil

**Keywords:** chromoblastomycosis, melanized fungi, immunological response, cell exhaustion, PD-1, PD-L1

## Abstract

The pathogenesis of chromoblastomycosis (CBM) is associated with Th2 and/or T regulatory immune responses, while resistance is associated with a Th1 response. However, even in the presence of IFN-γ, fungi persist in the lesions, and the reason for this persistence is unknown. To clarify the factors associated with pathogenesis, this study aimed to determine the polarization of the cellular immune response and the densities of cells that express markers of exhaustion in the skin of CBM patients. In the skin of patients with CBM, a moderate inflammatory infiltrate was observed, characterized primarily by the occurrence of histiocytes. Analysis of fungal density allowed us to divide patients into groups that exhibited low and high fungal densities; however, the intensity of the inflammatory response was not related to mycotic loads. Furthermore, patients with CBM exhibited a significant increase in the number of CD4^+^ and CD8^+^ cells associated with a high density of IL-10-, IL-17-, and IFN-γ-producing cells, indicating the presence of a chronic and mixed cellular immune response, which was also independent of fungal load. A significant increase in the number of PD-1^+^ and PD-L1^+^ cells was observed, which may be associated with the maintenance of the fungus in the skin and the progression of the disease.

## 1. Introduction

Chromoblastomycosis (CBM) is a neglected chronic subcutaneous disease commonly reported in tropical and subtropical regions of the world [1,2]. Etiological agents comprise approximately 41 species of saprophytic fungi distributed in 10 families of the order Chaetothyriales [3,4]; however, two main genera, *Fonsecaea* and *Cladophialophora*, have significant medical and epidemiological relevance [5,6]. In general, these fungi are found in the soil, are associated with plants or decaying organic matter, and are implanted in the skin through injuries caused by thorns, splinters, or other sharp objects [7].

Activation of immunity in CBM occurs shortly after inoculation of filamentous forms of the fungus in the skin. In this case, there will be activation of the local inflammatory response, allowing for leukocyte migration to the site of infection. These organisms are internalized by antigen-presenting cells (APCs) and quickly differentiate into muriform cells, which have a spherical shape and are pigmented due to a high melanin content [8].

Intracellular muriform cells are resistant to the microbicidal action of APCs [9,10], possibly due to the scavenger action of fungal melanin, which inhibits the microbicidal action of macrophages, as well as other phagocytic cells. Consequently, the fungus persists in the vertebrate host, potentializing the inflammatory response and triggering an adaptive immune response.

In this regard, lymphocytes can be found in the inflammatory infiltrate of patients with CBM [11]; however, until now, the T cell response in CBM has remained elusive. It is known that the severity of CBM is related to the different types of cellular immune responses (Th1, Th2, and Treg). In this regard, it was observed that patients with widely spread lesions associated with severe histopathological changes and a high fungal load presented a pronounced Th2 immune response, while patients with limited lesions and a low mycotic load produced a well-defined Th1 immune response [12]. Furthermore, peripheral blood mononuclear cells from patients with low CBM severity produced elevated levels of IFN-γ, but patients with high CBM severity produced large amounts of IL-10 [13], suggesting that the Th1 immune response is associated with resistance, while IL-10 is associated with persistence and susceptibility to infection. Thus, polarization of the acquired immune response will influence the macrophage responses at the site of infection [14], indicating that, when the Th1 immune response is activated, macrophages will be able to eliminate the parasitic forms of fungi; in contrast, if parasitic forms trigger a Th2 and/or Treg immune response, macrophages will be permissive to the multiplication of muriform cells, accounting for the chronification of infection [15].

The process of CBM chronification can cause functional changes in immune cells in the inflammatory infiltrate, such as cell exhaustion. This phenomenon occurs when T cells are constantly exposed to antigens or inflammatory signals during chronic infections or neoplasia, and it is characterized by progressive loss of effector functions, such as cytokine production, proliferative capacity, changes in metabolism, altered expression of transcription factors, and expression of high levels of inhibitory receptors [16,17]. These functional alterations occur due to the expression of the programmed cell death 1 molecule (PD-1) and its ligand (PD-L1) in the cell membrane of immune cells. PD-1 and PD-L1 are membrane glycoproteins expressed by cells of the immune system when consistently sensitized by an antigen [18,19], and they are considered inhibitory receptors that reduce cellular effector activities and are important in triggering cellular exhaustion, especially when PD-1 interacts with its ligands. In this sense, the PD-L1 glycoprotein, expressed primarily on the cell membrane of APCs, has been reported to bind to PD-1 present on the surface of T lymphocytes [20]. This interaction results in anergy of T cells and inhibition of macrophage activation [21], even in the presence of an inflammatory microenvironment associated with a Th1-type immune response, in which there is significant production of IFN-γ and nitric oxide [22,23,24], which in fact may be associated with the persistence of pathogenic microorganisms in the host.

These reports raise the question of which mechanisms would be involved in the permanence of the fungus and whether they were related only to the T cell response or also to its cellular exhaustion. Therefore, this article aims to quantify, in the histological sections of skin from CBM patients, the densities of iNOS-producing cells and Th1, Th2 and Th17 lymphocytes, as well as cells expressing PD-1 and PD-L1, and to associate these immunological markers with the intensities of the inflammatory process and infection. The data presented here demonstrate for the first time a substantial accumulation of cells expressing both markers of exhaustion (PD-1 and PD-L1), concomitant with a microbicidal immune response, marked by the presence of iNOS and IFN-γ. These data suggest that cell exhaustion may aid fungi persistence in humans with CBM.

## 2. Materials and Methods

### 2.1. Reagents and Dilutions

The primary antibodies rabbit polyclonal anti-human NOS2, recombinant rabbit monoclonal anti-human PD-1 (clone SJ01-91), recombinant rabbit monoclonal anti-human PD-L1 (clone RM320), mouse monoclonal anti-human CD4 (clone OKT-4), and mouse polyclonal anti-human IL-17A were obtained from ThermoFisher Scientific, Waltham, MA, USA. Rabbit polyclonal anti-human IFN-γ, rabbit polyclonal anti-human IL-4, rabbit polyclonal anti-human IL-10, and mouse monoclonal anti-human CD8 antibodies (clone C8/144B) were obtained from Abcam, Waltham, MA, USA. To perform immunohistochemistry, the following dilutions were used: anti-CD4 (1:10), anti-CD8 (1:25), anti-IFN-γ (1:125), anti-IL-4 (1:150), anti-IL-10 (1:700), anti-IL17A (1:500), anti-iNOS (1:200), anti-PD-1 (1:100), and anti-PD-L1 (1:200). The dilutions of each primary antibody were standardized using positive and negative controls according to the manufacturer’s recommendations. The Novolink Max polymer detection system kit (Leica, Wetzlar, Germany) and a diaminobenzidine (DAB) chromogenic substrate containing 3% H_2_O_2_ (DakoCytomation, Santa Clara, CA, USA) were used to reveal the reaction.

### 2.2. Patients, Skin Samples, and Ethical Aspects

In this study, previously collected skin biopsies of patients were used, which were obtained from the Ambulatório de Micologia do Departamento de Dermatologia do Hospital das Clínicas da Faculdade de Medicina da Universidade de São Paulo, São Paulo, SP, Brasil. Twenty-three samples from patients with a clinical diagnosis of CBM were analyzed, and these samples were collected before any antifungal treatment. These patients had a mean age of 61 years old (38–79 years), and they were all male. In addition, seven skin samples obtained from healthy individuals who underwent plastic surgery were included as controls. This study was approved by the Research Ethics Committee of the São Paulo State University (n. 55998421.2.0000.5411.) and the Hospital das Clínicas da Faculdade de Medicina da Universidade de São Paulo (n. 55998421.2.3001.0068).

### 2.3. Evaluation of the In Situ Inflammatory Response

Histological sections of the skin were obtained, deparaffinized in xylene, hydrated in two passages in absolute alcohol and one passage in 70% alcohol, and finally washed in two passages of distilled water. Further, the histological sections were stained with hematoxylin and, after washing in distilled water, stained with eosin. The slides were then quickly immersed in distilled water, dehydrated in one pass of 70% alcohol and two washes in absolute alcohol, cleared in one pass of xylene, and then mounted in synthetic balm.

To evaluate the inflammatory response in skin tissue lesions, a semiquantitative comparative analysis was performed according to the modified method of Ridley and Ridley [25]. The results were scored based on the intensity of each tissue parameter (inflammatory process, presence of mono- and polymorphonuclear cells, lymphocytes, and plasma cells) observed using a 40× objective as follows: (score 0) negative; 1–50 cells (score: 1) discrete; 50–100 cells (score: 2) moderate; and more than 100 cells (score: 3) intense. Semiquantitative analysis was performed in 10 random fields in the inflammatory infiltrate region by three different researchers.

Fungal density and the presence of giant cells were quantitatively evaluated in HE-stained histological sections; in this case, at least 10 random fields of the inflammatory infiltrate of each case were recorded using an optical microscope coupled to a microcomputer using AxionVision software, version 4.3; the number of muriform forms was quantified, and the fungal density (d) was calculated using the ratio:

d = N/A,

where N is number of muriform or giant cells, and A is the area of each recorded histological image, which was 0.0357 μm^2^.

### 2.4. Immunostaining for Cytokines, iNOS Enzyme, CD4, CD8 and Immunological Factors Related to Cell Exhaustion

Histological sections were added to slides previously treated with organosilane (Sigma, Rockville, MD, USA), and then biological materials were dewaxed in xylene at room temperature for 15 min, followed by another passage in xylene for 2 min. They were then hydrated in two passages of absolute alcohol for 2 min, one passage in 70% alcohol for 2 min, and finally two quick passages in distilled water. The endogenous peroxidase was then blocked by immersing the histological sections in a solution of 3% hydrogen peroxide (H_2_O_2_) 10 times, 3 min each, at room temperature in the dark.

After this step, antigen retrieval was performed using different retrieval buffers. To immunostain IFN-γ, IL-4, IL-10, iNOS, CD8, PD-1, and PD-L1 buffer composed of 10 mM sodium citrate buffer was used with a pH of 6.0. To immunostain IL-17A, 1 mM EDTA buffer with a pH of 8.0 was used as a retrieval buffer; and to stain CD4, a retrieval buffer, constituted of 10 mM Tris base plus 1 mM EDTA with a pH of 9.0, was used. In all cases, antigen retrieval was performed in a water bath at 96–99 °C for 30 min. After this period, when the citrate buffer reached 55 °C, the histological section was incubated with 6% skim milk powder diluted in PBS (*w*/*v*) (Molico^®^—Nestlé) to block nonspecific bindings for 60 min at 37 °C.

All primary antibodies were added to the histological sections in sufficient quantities to cover the skin sections. All antibodies were diluted in a PBS solution containing 1% bovine albumin (PBS-BSA). As a negative control for the reaction, histological sections were incubated only with diluent solution (PBS-BSA). Histological sections were incubated for 18 h in a humid chamber at 4 °C. After this period, the histological skin sections were washed three times with 0.15 M buffered saline solution containing 0.05% Tween 20 (PBS-T) for 5 min each. Histological sections were then incubated with rabbit anti-mouse post-primary antibody (Novolink, Newcastle, UK) for 30 min at 37 °C and then washed with PBS-T (three times, 5 min each). The sections were incubated with anti-rabbit polymer-HRP (Novolink, UK) for 30 min at 37 °C and then washed with PBS-T (three times, 5 min each). DAB (DakoCytomation, Santa Clara, CA, USA) was added to the histological sections and incubated for a maximum period of 5 min at room temperature. Subsequently, the slides were washed twice with distilled water and counterstained with Harris hematoxylin for 2 min. Histological sections were washed in distilled water, followed by dehydration in 70% alcohol for 2 min, two washes in absolute alcohol for 2 min, and one wash in xylene for 2 min. The sections were dried at room temperature and then mounted with resin and a glass coverslip.

### 2.5. Quantitative Analysis

For this analysis, at least 10 random fields of the inflammatory infiltrate of each skin histological section (40× objective) were photographed using an optical microscope coupled to a microcomputer. Images were obtained using AxionVision software, version 4.3 (Carl Zeiss Microscopy, White Plains, NY, USA). The immunostained cells were quantified considering the differential staining in the histological sections, and cell densities were estimated as detailed in Section 2.3. A semi-quantitative evaluation was performed by three different researchers.

### 2.6. Statistical Analysis

Data obtained from the skin of patients with CBM were compared with data from normal skin. All statistical analyzes were performed using GraphPad Prism software, version 8. Based on the nature of the nonparametric distribution of the data obtained, the Mann–Whitney test was used, and when *p* < 0.05, the differences between the groups were considered significant.

Linear regressions were performed to verify whether there was a relationship between cell exhaustion markers (PD-1 and PD-L1) and the age of the patients in this study.

## 3. Results

### 3.1. Inflammatory Infiltrate and Muriform Cells

**Figure 1:**
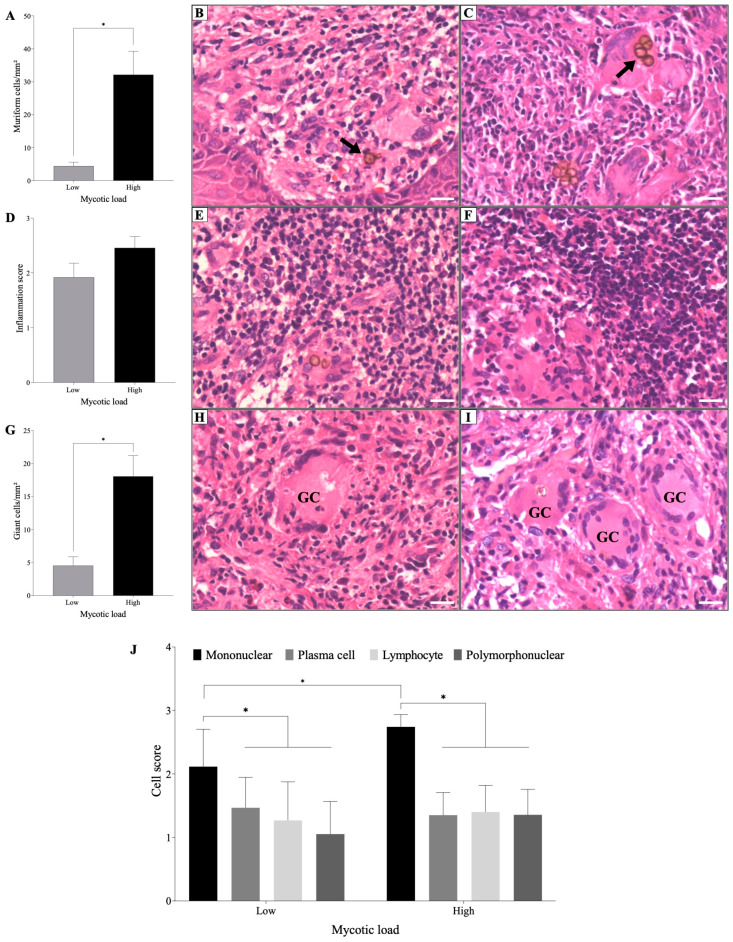
Characterization of the histological changes in the skin of patients with CBM. (**A**) Fungal load in patients with high and low densities of muriform cells. (**B**,**C**) Demonstration of muriform cells in patients with low (**B**) and high (**C**) fungal densities. (**D**) Semiquantitative analysis of the inflammatory infiltrate and demonstration of the infiltrate in patients with low (**E**) and high (**F**) mycotic loads. (**G**) Densities of giant cells in patients with low (**H**) and high (**I**) mycotic loads. (**J**) Semi quantitative analysis characterizing the main cell types in the inflammatory infiltrate in patients with low and high mycotic densities. Skin sections were stained with hematoxylin and eosin (40× objective, scale bar = 20 μm). The arrows indicate muriform cells (**B**,**C**). The initials ‘GC’ indicate giant cells (**H**,**I**). * *p* < 0.05 indicates a significant difference between the groups. The results are expressed as the mean ± standard deviation.

Quantitative analysis of muriform cells revealed that the mycotic load of the skin was classified into two groups (*p* < 0.05): one with patients with a low mycotic load (≤15 muriforms/mm^2^—Low) and the other with patients a high mycotic load (≥15 muriforms/mm^2^—High), as observed in Figure 1A–C. In this case, 52.7% (12/23) of the patients were divided into the “low group”, while 47.8% (11/23) were in the “high group”.

Regarding the inflammatory response, it was observed that it was distributed mainly in the dermis of all patients, and the intensity was moderate in groups exhibiting high and low mycotic loads (Figure 1D–F). In the inflammatory infiltrate, 100% (23/23) of the samples presented giant cells, the density of which was correlated with the fungal load (*p* < 0.05) (Figure 1G–I); thus, patients with low fungal density had a small number of giant cells, while patients with high fungal load had high density of giant cells (Figure 1G).

The characterization of the inflammatory infiltrate allowed for the identification of the presence of polymorpho- and mononuclear leukocytes, lymphocytes, and plasma cells, with a significant predominance (*p* < 0.05) of mononuclear cells (mainly histiocytes) in all patients, suggesting that, independent of the fungal load, the inflammatory infiltrate was similar in patients with CBM. However, patients with a high fungal load had significant infiltration of mononuclear cells compared to the group with a low fungal load (Figure 1J).

### 3.2. Evaluation of the Cellular Immune Response (In Situ)

**Figure 2:**
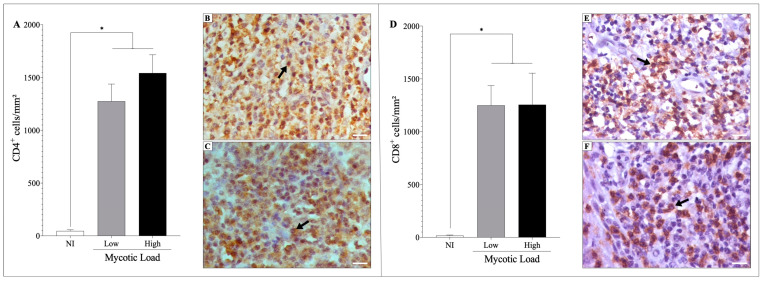
Lymphocyte populations were analyzed by immunohistochemistry (IHC) in the skin histological section of patients with CBM and in the skin of healthy individuals (NI). (**A**) Density of CD4^+^ cells in patients with low (**B**) and high (**C**) fungal densities. (**D**) Density of CD8^+^ cells in patients with low (**E**) and high (**F**) fungal densities. Images of IHC reactions were recorded with a 40× objective; scale bar = 20 μm. Arrows indicate CD4^+^ (**B**,**C**) and CD8^+^ (**E**,**F**) cells. * *p* < 0.05 indicates a significant difference between the groups. The results are expressed as the mean ± standard deviation.

Immunostaining of the lymphocyte subsets revealed that the density of the CD4 (Figure 2A–C) and CD8 (Figure 2D–F) T cell subsets increased compared to the non-infected control group (*p* < 0.05); however, no differences were observed between patients with low and high mycotic loads.

**Figure 3:**
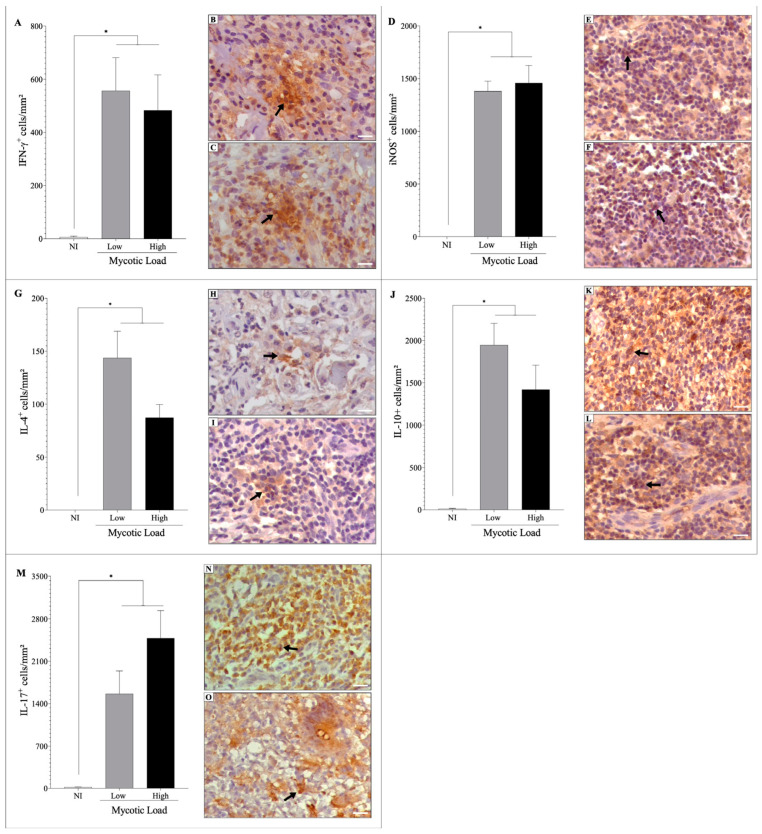
The cellular immune response was analyzed by immunohistochemistry (IHC) in the skin histological section of patients with CBM and in the skin of healthy individuals. (**A**) Density of IFN-γ^+^ cells in patients with low (**B**) and high (**C**) fungal densities. (**D**) Density of iNOS^+^ cells in patients with low (**E**) and high (**F**) fungal densities. (**G**) Density of IL-4^+^ cells in patients with low (**H**) and high (**I**) fungal densities. (**J**) Density of IL-10^+^ cells in patients with low (**K**) and high (**L**) fungal densities. (**M**) Density of IL-17^+^ cells in patients with low (**N**) and high (**O**) fungal densities. Images of IHC reactions were recorded in 40× objective; scale bar = 20 μm. Arrows indicate IFN-γ^+^ (**B**,**C**), iNOS^+^ (**E**,**F**), IL-4^+^ (**H**,**I**), IL-10^+^ (**K**,**L**), and IL-17^+^ (**N**,**O**) cells. * *p* < 0.05 indicates a significant difference between the groups. The results are expressed as mean ± standard deviation.

Immunostaining of inflammatory mediators showed that the skin of patients with CBM exhibited a significant increase (*p* < 0.05) in the densities of IFN-γ^+^ (Figure 3A–C), iNOS^+^ (Figure 3D–F), IL-4^+^ (Figure 3G–I), IL-10^+^ (Figure 3J–L), and IL-17^+^ (Figure 3M–O) cells compared to the uninfected group (NI). However, the densities of these cells were similar in groups of patients with low or high fungal loads. Furthermore, all analyzed samples were observed to be positive for such immunological markers.

**Figure 4:**
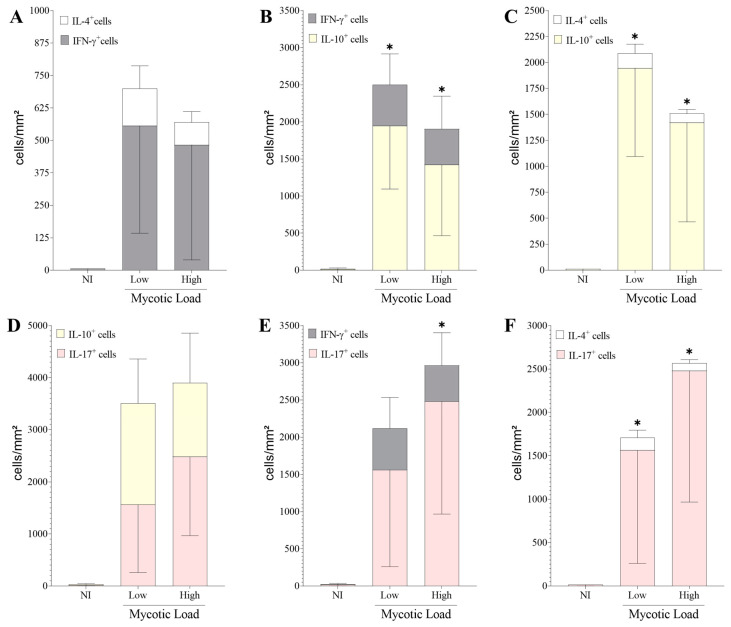
Comparisons between immunological responses of IFN-γ^+^ and IL-4^+^ cells (**A**); IFN-γ^+^ and IL-10^+^ cells (**B**); IL-4^+^ and IL-10^+^ cells (**C**); IL-10^+^ and IL-17^+^ cells (**D**); IFN-γ^+^ and IL-17^+^ cells (**E**); IL-4^+^ and IL-17^+^ cells (**F**). * *p* < 0.05 indicates a significant difference between cell densities in the same experimental group. The results are expressed as mean ± standard deviation.

To compare the dichotomy of immune responses, the relationships among cells producing IL-4/IFN-γ (Figure 4A), IFN-γ/IL-10 (Figure 4B), IL-4/IL-10 (Figure 4C), IL-10/IL-17 (Figure 4D), IFN-γ/IL-17 (Figure 4E), and IL-4/IL-17 (Figure 4F) were analyzed. As noted above, fungal burden was not a decisive factor in stimulating a specific immune response; however, the presence of muriform cells in patients with CBM was observed to induce a predominant and significant increase (*p* < 0.05) in an immunoregulatory response mediated primarily by IL-10 and IL-17 compared to the cytokines IFN-γ (Figure 4B,E) and IL-4 (Figure 4C,F), suggesting that the disease progresses with a predominance of a regulatory T and Th17 immune response over Th1 and Th2 immune responses.

### 3.3. Immunostaining of the Cell Exhaustion Markers

**Figure 5:**
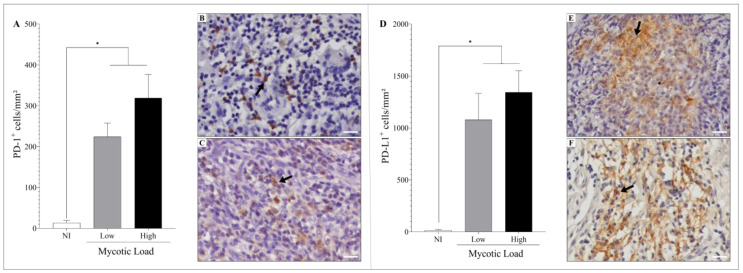
Cell exhaustion was studied in the histological sections of patients with CBM by immunohistochemistry. (**A**) Density of PD-1^+^ cells in patients with low (**B**) and high (**C**) fungal densities. (**D**) Density of PD-L1^+^ cells in patients with low (**E**) and high (**F**) fungal densities. The images were recorded with a 40× objective; scale bar = 20 μm. Arrows indicate PD-1^+^ (**B**,**C**) and PD-L1^+^ (**E**,**F**) cells. * *p* < 0.05 indicates a significant difference between the groups. The results are expressed as mean ± standard deviation.

Regarding the markers of exhaustion, it was observed that all samples had positivity for PD-1 and PD-L1 (Figure 5); however, patients with CBM exhibited a significant increase in the density of PD-1^+^ and PD-L1^+^ cells (*p* < 0.05) compared to healthy individuals (Figure 5A–F). Furthermore, the groups presenting low or high fungal loads presented similar densities of PD-1^+^ and PD-L1^+^ cells, as observed in Figure 5A,D.

There is a consensus regarding a positive correlation between aging and the expression of PD-1 and PD-L1 [26]. Therefore, correlation analyses were performed to verify whether the increase in the expression of these markers was associated with the age of the patients studied.

**Figure 6:**
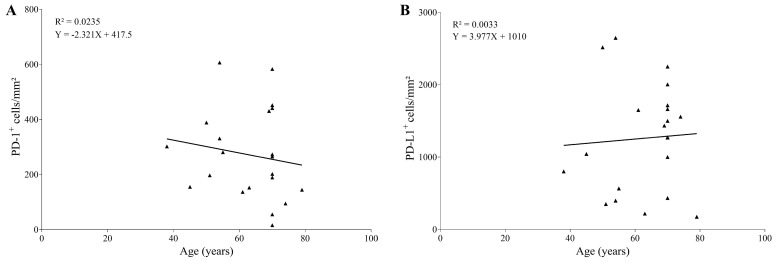
Correlation between the age of the patients and the densities of cells expressing the exhaustion markers PD-1 (**A**) or PD-L1 (**B**).

Linear regression between cell densities of PD-1^+^ or PD-L1^+^ versus patient age (Figure 6A,B) showed correlations between variables (R^2^ = 0.0235 for PD-1; and R^2^ = 0.0033 for PD-L1), suggesting that fungal infection results in an altered expression pattern of these cell exhaustion markers; therefore, the existing correlation in human physiology is lost.

## 4. Discussion

Among the neglected tropical diseases caused by saprophytic melanized fungi, CBM is one of the most prevalent; its etiological agents live in association with the plants that agricultural and logger workers manipulate during their work, and after an injury, they access the skin [4,27,28]. After infection, the fungi differentiate into muriform cells within phagocytes, becoming spherical and pigmented [8,29]. They resist microbicidal response phagocytes, and as a consequence, parasitic forms of fungi will survive and multiply in the vertebrate host.

In the present study, quantification of muriform cells allowed us to divide patients enrolled in this study into groups exhibiting low mycotic loads (low) or high mycotic loads (high), suggesting that patients with a high fungal load presented a more severe clinical condition, as demonstrated in studies conducted in patients with CBM [12,30] and BALB/c mice infected with *F. pedrosoi* [31]. Although works have associated disease severity and intensity of inflammatory infiltrate with a high mycotic load, in this article, no association was observed, as the intensity of the inflammatory response was similar in groups of patients with low or high mycotic loads.

Furthermore, the composition of the inflammatory infiltrate was similar in patients with low or high fungal densities, basically composed of polymorphonuclear and mononuclear cells, such as histiocytes, plasma cells, and lymphocytes. In addition, the main cell type in the inflammatory infiltrate was histiocytes, which appeared in greater densities in patients with high parasitic forms of fungi than in patients with low loads of fungi, possibly because a larger number of muriform cells attract more monocytes to the site of infection [11]. Furthermore, it was verified that these cells fused, creating multinucleated giant cells containing or not muriform cells, as well as cellular debris in the cytoplasm of these cells [32,33]. It is possible that, in human CBM, the presence of giant cells in the inflammatory infiltrate is a common event coordinated by the immune system that aims to eliminate muriform cells, as observed in infections caused by *Fonsecaea nubica* [34], *Veronae botryosa* [35], and other species of fungi [36,37,38]. Thus, in the present manuscript, the presence of giant cells at the site of infection should be associated with an attempt to contain parasitic forms of the fungus. Despite the microbicidal activity of giant cells, muriform cells possibly evaded giant cell responses, considering the progression of the disease.

In this study, the lymphocytes present in the inflammatory infiltrate of the patients comprised the CD4 and CD8 subsets. Naive CD4^+^ T lymphocytes can be differentiated according to some stimuli into subsets, such as Th1, Th2, Th17, and regulatory T cells, which in turn produce mainly IFN-γ, IL-4, IL-17, and IL-10 cytokines, respectively [39]. In the present study, a high density of CD4 lymphocytes was detected in the inflammatory infiltrate of patients with CBM, which was correlated with high production of IFN-γ and IL-17, suggesting that both Th1 and Th17 immune responses were activated to eliminate parasitic forms of fungi. In addition, the efflux of such cells did not depend on the density of muriform cells. To eliminate intracellular fungi, Th1 and Th17 immune responses must be activated since IFN-γ will activate macrophages to a fungicidal state, and IL-17 plays an important role in facilitating neutrophil infiltration and the production of antifungal peptides [40,41], which are associated with resistance to fungal infection [42,43,44,45,46,47].

Furthermore, the cytokine IFN-γ, secreted mainly by CD4^+^ lymphocytes, stimulates the expression of the iNOS enzyme in phagocytic cells, leading to the production of NO metabolite that has significant microbicidal activity [48,49]. In fact, in the present study, it was verified that patients with CBM produced IFN-γ and iNOS; therefore, immunological cells from the patients studied herein would be capable of controlling intracellular infections, including intracellular fungi [13]. However, the fungi persisted at the site of infection and evaded a Th1 immune response, causing progressive disease. Some studies have pointed to an important property of fungal melanin in scavenging reactive nitrogen and oxygen species produced by APC [50], which may explain the persistence of parasitic forms in the skin.

Despite an immune response associated with macrophage activation, Th2 and T regulatory immune responses, associated with IL-4 and IL-10 production, were observed; these immunological cells in the inflammatory infiltrate are possibly capable of inhibiting the Th1 immune response. In fact, IL-4 counterbalances the immunological property of IFN-γ by restricting the conversion of NO into macrophages [51]. In intracellular bacterial infections, IL-4 has been linked to susceptibility and disease progression, allowing *Staphylococcus aureus* growth [52]. IL-4 is also related to the persistence of *Candida albicans* in macrophages in vitro [53] and the progression of aspergillosis in mice [54,55]; therefore, IL-4 may have contributed to the chronicity of CBM in the patients studied here. In addition to IL-4, the cytokine IL-10 inhibits the production of IFN-γ and reduces the expression of the major histocompatibility complex and co-stimulatory molecules [39]. Therefore, these data suggest that both cytokine-producing cells may inhibit the interaction between lymphocytes as phagocytic cells, allowing for the progression of CBM [50,56].

Furthermore, in this study, it was observed that the density of IL-10^+^ cells was approximately 200 times greater than the density of IFN-γ^+^ cells and 1200 times greater than the density of IL-4^+^ cells. In addition, an equivalent proportion of IL10^+^ and IL17^+^ cells was observed. Therefore, these data indicated that, although patients have mounted a response associated with fungal elimination with the participation of giant cells, iNOS^+^ cells, Th1, and Th17 cells, an excessive regulatory immune response also may impair the immunologic potential of these cells. In fact, it has already been observed that, in an inflammatory microenvironment with a predominance of IL-10, a microbicidal response will be abrogated [57], even with a significant accumulation of giant and iNOS-producing cells, which have expressive resources to eliminate intracellular pathogens in the inflammatory infiltrate.

Furthermore, CD8^+^ cells were also observed in the inflammatory infiltrate of patients with CBM studied here. These cells induce a highly regulated apoptosis process in target cells and can control intracellular infections, as described in experimental animals infected with *Candida albicans* [58], *Cryptococcus neoformans* [59], and *Pneumocytis carinii* [60], and these cytolytic T cells are activated when hosts exhibit a compromised or insufficient response to CD4^+^ T cells. Considering that CBM patients had a mixed immune response that could not be exclusively associated with microbe elimination, the influx of CD8^+^ cells at the site of infection may, at least, be associated with partial control of muriform cells [43,44].

Persistence of an infection, in general, leads to the chronification of the inflammatory response, which in turn can trigger a process known as immunological exhaustion [61]. In the present study, the molecule PD-1 and its ligand PD-L1, associated with cell exhaustion, were analyzed. PD-1 is expressed mainly by lymphocytes, while its ligand, PD-L1, can be expressed by macrophages and dendritic and polymorphonuclear cells. Therefore, the interaction between PD-1^+^ lymphocytes and PD-L1^+^ APC can inhibit antigen presentation, production of pro-inflammatory cytokines, and monocyte proliferation and differentiation to the M1 subset [21,61], allowing for the multiplication of pathogens [19,62]. In the present study, a large number of PD-1^+^ and PD-L1^+^ cells was observed in the skin of patients with chromoblastomycosis, in fact suggesting that these molecules control the activity of the immune response, characterized by the presence of Th1, Th2, Th17, and T-reg cells in the site of infection.

In addition to this process, it has been observed [63] that the PD-1/PD-L1 pathway may induce CD8^+^ T lymphocytes to eliminate M1 macrophages by cytotoxicity [63]. This subset of macrophages has significant microbicidal activity [64]; therefore, the accumulation of this type of cell is important at the site of infection [65]. Thus, the presence of CD8^+^ cells associated with elevated expression of PD-1 and PD-L1 markers in the skin of patients with CBM may be linked not only to the control of fungi but also to the elimination of M1 macrophages.

Although this report demonstrated for the first time the participation of PD-1^+^ and PD-L1^+^ cells in the skin of patients with CBM, the same markers of cell exhaustion have been described as factors associated with disease progression in infections caused by viruses [64,66], bacteria [67,68,69,70,71,72], protozoans [73,74], and helminths [75,76,77]. In these infections, a correlation was observed between the intensity of the infection and the expression of cell exhaustion markers. In in vitro fungal infections caused by *Aspergillus fumigatus* [78,79] and in mice infected with *Candida auris* [80], an increase in the expression of cellular exhaustion factors was observed, and the number of PD-L1^+^ cells was positively correlated with the systemic fungal load [80]. Therefore, the presence of PD-1^+^ and PD-L1^+^ cells in the skin of patients may have a pathogenic role during infection.

In fact, experimental studies have shown that, when the exhausted phenotype is reversed in infectious diseases [73,74], animals present significant improvement, suggesting that exhaustion markers play a pivotal role in modulating immune responses. Recently, it was verified that, by blocking PD-1 and PD-L1, mice survived after fungal sepsis caused by *Candida albicans* [81]. Furthermore, PD-1^−/−^ mice infected with *Histoplasma capsulatum* survived after infection, while WT mice expressed high levels of PD-L1 and died after the 25th day of infection [82]. Such reports support the idea that immunotherapy based on the use of anti-PD-1 and anti-PD-L1 monoclonal antibodies, for example, may be used in the future for the treatment of CBM, as the inflammatory infiltrate observed in all cases studied here exhibited high densities of PD-1^+^ and PD-L1^+^ cells.

Additionally, it is recognized that aging leads to increased expression of PD-1 and PD-L1, which in turn are related to cellular senescence [83,84,85] or the accumulation of memory T cells [86,87]. To verify whether there would be an impact on the age of the patients in the presence of cells with the exhaustion phenotype, a correlation test was performed. In this specific situation, it was observed that age was not a decisive factor for increased expression of PD-1 or PD-L1, but the presence of the fungus modified a physiological phenomenon observed during aging.

Finally, the present study suggests that the inflammatory response in CBM is not modulated according to the fungal burden; therefore, patients with a low or high number of muriform cells exhibited a similar inflammatory response, suggesting that the fungus has high immunogenic potential. Despite the difference in the fungal loads, some factors, such as fungal species, time of evolution, and clinical form of CBM, are also significant in determining the inflammatory process. Furthermore, our results highlight that the presence of IL-4^+^ and IL-10^+^ cells should contain the microbicidal activity of giant cells, as well as the effector functions of the Th1+ and Th17+ cells in controlling the spread of the fungus through the skin. Finally, a chronic inflammatory response observed in all patients must lead to cell exhaustion, mediated by the expression of PD-1 and PD-L1 molecules, in turn leading to fungal persistence and infection progression.

## Data Availability

The data presented in this study are available on request from the corresponding author.
